# Identifying small-effect genetic associations overlooked by the conventional fixed-effect model in a large-scale meta-analysis of coronary artery disease

**DOI:** 10.1093/bioinformatics/btz590

**Published:** 2019-07-27

**Authors:** Lerato E Magosi, Anuj Goel, Jemma C Hopewell, Martin Farrall

**Affiliations:** Wellcome Centre for Human Genetics; Division of Cardiovascular Medicine, Radcliffe Department of Medicine; Wellcome Centre for Human Genetics; Division of Cardiovascular Medicine, Radcliffe Department of Medicine; Nuffield Department of Population Health, University of Oxford, Oxford, UK; Wellcome Centre for Human Genetics; Division of Cardiovascular Medicine, Radcliffe Department of Medicine

## Abstract

**Motivation:**

Common small-effect genetic variants that contribute to human complex traits and disease are typically identified using traditional fixed-effect (FE) meta-analysis methods. However, the power to detect genetic associations under FE models deteriorates with increasing heterogeneity, so that some small-effect heterogeneous loci might go undetected. A modified random-effects meta-analysis approach (RE2) was previously developed that is more powerful than traditional fixed and random-effects methods at detecting small-effect heterogeneous genetic associations, the method was updated (RE2C) to identify small-effect heterogeneous variants overlooked by traditional fixed-effect meta-analysis. Here, we re-appraise a large-scale meta-analysis of coronary disease with RE2C to search for small-effect genetic signals potentially masked by heterogeneity in a FE meta-analysis.

**Results:**

Our application of RE2C suggests a high sensitivity but low specificity of this approach for discovering small-effect heterogeneous genetic associations. We recommend that reports of small-effect heterogeneous loci discovered with RE2C are accompanied by forest plots and standardized predicted random-effects statistics to reveal the distribution of genetic effect estimates across component studies of meta-analyses, highlighting overly influential outlier studies with the potential to inflate genetic signals.

**Availability and implementation:**

Scripts to calculate standardized predicted random-effects statistics and generate forest plots are available in the *getspres* R package entitled from https://magosil86.github.io/getspres/.

**Supplementary information:**

Supplementary data are available at *Bioinformatics* online.

## 1 Introduction

The conservative nature of the traditional random-effects model (RE), which assumes the presence of heterogeneity under the null, has contributed to the dominance of fixed-effect (FE) meta-analysis methods in the discovery of small-effect variants (per-allele disease odds ratios <1.2 or trait variance <0.2%) (Bush and Moore, 2012; Yang *et al.*, 2011) even at heterogeneous loci. A modification of the traditional random-effects method, RE2, was designed to detect genetic associations both in the presence and absence of heterogeneity, to provide an opportunity to identify small-effect heterogeneous variants that might go unnoticed in a FE meta-analysis (Han and Eskin, 2011). Most users of the RE2 random-effects method employed it to refine associations at significant and suggestive genetic signals identified in FE meta-analyses (Sapkota *et al.*, 2015, 2017; Wyss *et al.*, 2018); in its latest iteration, RE2C (Lee *et al.*, 2017) it reports a subset of variants detected by RE2 where $P_{\mathrm{RE}2}\leq \;P_{\mathrm{FE}.}$ The RE2C update is intended to have a broad application beyond the augmentation of summary association *P*-values of variants identified in FE meta-analysis to the discovery of additional and potentially novel loci. The RE2 and by extension RE2C random-effects method’s power advantage over traditional fixed and random-effects meta-analysis models is partly attributable to a relaxed null hypothesis, which assumes homogeneity of genetic effects under the null and thereby provides a greater contrast between the null and alternative hypotheses$\;\left(H_{0}:\;\mu \;=\;0\;\mathrm{and}\;\tau^{2}=0\;\mathrm{versus}\;H_{1}:\;\mu \;\neq \;0\;\mathrm{or}\;\tau^{2}>0;\;\mathrm{asymptotically}\right)$.

Heterogeneity of genetic effects might arise from biologically relevant differences among contributing studies in a meta-analysis, such as diverse: ancestries, linkage disequilibrium patterns, sub-phenotypes, ages-of disease onset, family-history of disease or gender. Alternatively, differences in the direction and/or size of genetic effect-estimates among participating studies in a meta-analysis could reflect genotyping error or population structure (i.e. local admixture), where, for example, the average genetic effect estimate at a variant of interest is inflated by a few outlier studies showing outsized effects while the majority of study effects are marginal. Heterogeneity at individual variants can be explored through forest plots and the calculation of standardized predicted random-effects (SPREs), while heterogeneity patterns across multiple variants can be conveniently inspected through the calculation of M statistics (Magosi *et al.*, 2017). Notably, SPREs are precision weighted residuals that indicate the direction and extent with which individual studies in a meta-analysis deviate from the average genetic effect (Harbord and Higgins, 2008; Magosi *et al.*, 2017), and can be a useful quantitative indicator of whether the average genetic effect at a variant of interest might be unduly influenced by outlier studies showing extreme effects.

In this report, we revisit the CARDIoGRAMplusC4D meta-analysis (60 801 cases and 123 504 controls) of coronary artery disease (CAD) with the RE2C random-effects method, to search for additional CAD loci potentially masked by heterogeneity in the primary FE meta-analysis.

## 2 Materials and methods

### 2.1 GWAS datasets

#### 2.1.1 CARDIoGRAMplusC4D

Summary data (i.e. logistic regression coefficients and their corresponding standard errors) were collated from 48 genome-wide association studies of coronary disease risk that comprised individuals from 6 different ancestry groups including: African (*n* = 1) and Hispanic American (*n* = 1), East (China and Korea, *n* = 3) and South (India and Pakistan, *n* = 4) Asian, Middle Eastern (Lebanese, *n* = 1) and European (*n* = 38); meta-analysis was conducted for a set of ∼ 9 million variants with minor allele frequencies >0.005 (CARDIoGRAMplusC4D Consortium, 2015). Design details of each participating CARDIoGRAMplusC4D study are summarized in Supplementary Table S1; the coronary disease phenotype included patients with an inclusive CAD diagnosis (e.g. myocardial infarction, acute coronary syndrome, chronic stable angina or coronary stenosis >50%). Study-level genomic correction (Devlin and Roeder, 1999) was applied to each study to minimize false positives induced by inflated association test statistics. Variant effect-size estimates (β coefficients scaled as log_e_(odds ratios) from an additive-effects-only association model) in each study were aligned such that the same risk allele was compared across the studies assembled in the meta-analysis. The studies contributing to the CARDIoGRAMplusC4D study obtained ethical approval from the ethics committees of the respective medical faculties, and informed consent was obtained from all participants, summary genetic association data were anonymously meta-analysed and reported here. Membership of the CARDIoGRAMplusC4D Consortium is provided in the Supplementary Text S1. Requests for access to the summary statistics are coordinated by the CARDIoGRAMplusC4D Steering Committee (www.cardiogramplusc4d.org).

#### 2.1.2 UK Biobank

The UK Biobank study (UKBB) is a large-scale prospective study of over half a million participants commissioned to assemble comprehensive data on genotypic, socio-demographic, lifestyle and environmental factors with the aim of developing better strategies for the prevention, diagnosis and treatment of common diseases (Sudlow *et al.*, 2015) such as cardiovascular disease (Littlejohns *et al.*, 2019). Data from an interim release of GWAS genotypes for 296 525 participants were previously merged and analysed with clinical phenotype data that identified 34 541 cases of coronary heart disease and 261 984 controls from England, Scotland and Wales aged 45–69 years (van der Harst and Verweij, 2018). Coronary disease case status was assigned to prevalent and incident cases of myocardial infarction, acute coronary syndromes and associated therapeutic interventions (e.g. revascularization). Association summary statistics (β coefficients scaled as log_e_(odds ratios) and associated standard errors from an additive-effects-only logistic regression association model) from this analysis were downloaded from the www.cardiomics.net server. Design details of the UK Biobank participants to compare with the CARDIoGRAMplusC4D cohorts are included in Supplementary Table S1.

### 2.2 RE2 and RE2C meta-analysis

Genetic association meta-analyses are typically performed under a RE when the objective is both to estimate a summary effect (i.e. average genetic effect) across studies in a meta-analysis and measure the amount of heterogeneity. Consider a meta-analysis comprising *S* studies (s = 1, 2, 3,…, S) where the genetic effect-size estimate and corresponding standard error of a variant of interest were obtained via regression modelling in each study, and the average genetic effect estimate, $\hat{\mu }$ calculated as the inverse-variance weighted mean of the individual study effects:

(1) $$\hat{\mu }=\;\frac{\sum_{s=1}^{S}w_{s}y_{s}}{\sum_{s=1}^{S}w_{s}}\;$$

where $y_{s}$ represents the study effect-size estimate in the *s*th study, and $w_{s}$ denotes the weight assigned to the *s*th study, which can be calculated as $w_{s}=\frac{1}{\left(\sigma_{s}^{2}+\;{\hat{\tau }}^{2}\right)}.\;$Notably, $\sigma_{s}^{2}$ and ${\hat{\tau }}^{2}$ represent sampling variance and heterogeneity, respectively.

#### 2.2.1 Traditional RE

The traditional RE tests the null hypothesis that the average genetic effect, μ is zero that is, $H_{0}:\mu \;=\;0\;\mathrm{versus}\;H_{1}:\mu \;\neq \;0$, and its summary association test statistic under the null is given by, $Z_{\mathrm{RE}}^{2}={\left(\frac{\hat{\mu }}{\mathrm{SE}(\hat{\mu })}\right)}^{2}\;∼\;\chi_{1}^{2}$ (asymptotically) (Neupane *et al.*, 2012).

#### 2.2.2 Contemporary random-effects model (RE2)

In contrast to the traditional RE which assumes the presence of heterogeneity under the null the RE2 model tests the null hypothesis that the average genetic effect is zero and there is no heterogeneity; that is, $H_{0}:\mu \;=\;0\;\mathrm{and}\;\tau^{2}=0\;\mathrm{versus}\;H_{1}:\mu \;\neq \;0\;\mathrm{or}\;\tau^{2}>0$ (asymptotically) (Han and Eskin, 2011; Neupane *et al.*, 2012). The summary association test statistic (or likelihood ratio test statistic) for the RE2 model under the null is denoted by:

(2) SRE2= ZRE (new)2= −2log(λ)        = −2log(L0(0,0)L1(µ^,^τ 2)),

and approximates a 50:50 mixture of $\chi_{1}^{2}$ and $\chi_{2}^{2}$ asymptotically in meta-analyses with larger numbers of studies. For meta-analyses with fewer studies (2–50), Han and Eskin provide tabulated RE2 *P*-values corrected for small sample-size based on the assumption that the studies are equally weighted (i.e. same sample-size). The asymptotic RE2 summary association *P*-value is denoted by:

$${P^{*}}_{\mathrm{RE}2}=0.5\cdot \;P\left(\chi_{\left(1\right)}^{2}\;\geq \;S_{\mathrm{RE}2}\right)+0.5\;\cdot \;P\left(\chi_{\left(2\right)}^{2}\;\geq \;S_{\mathrm{RE}2}\right),$$

after a correction for small samples, the RE2 summary association *P*-value is given by:

$$P_{\mathrm{RE}2}=\;\lambda \left(N,S_{\mathrm{RE}2}\right)\cdot \;{P^{*}}_{\mathrm{RE}2},$$

where $\lambda \left(N,S_{\mathrm{RE}2}\right)$ is the small-sample correction factor (Lee *et al.*, 2017).

#### 2.2.3 Updated RE2 model (RE2C)

The RE2C approach is an adaptation of the RE2 model designed to: (i) facilitate discovery of small-effect heterogeneous variants and (ii) minimize redundancy between genetic variants identified by the FE and RE2 models; as it is commonplace to perform an FE analysis prior to a random-effects analysis when conducting genetic association meta-analyses. To reduce redundancies between RE2 and FE analyses the RE2C approach partitions summary association *P*-values produced by the RE2 model into two groups assigning variants with RE2 *P*-value ≤ FE *P*-value the RE2 summary association statistic, $S_{\mathrm{RE}2}$ and zero otherwise (Lee *et al.*, 2017):

(3) SRE2C=SRE2 if PRE2≤PFE0 if PRE2>PFE . 

In contrast to the RE2 summary association statistic the RE2C statistic, $S_{\mathrm{RE}2C}$ does not approximate a ‘well-known’ asymptotic distribution; to calculate RE2C *P*-values the RE2 summary association statistic is decomposed into two component statistics, the first, $S_{\mathrm{FE}}$ is equal to the square of the FE summary association statistic, $Z_{\mathrm{FE}}^{2}$ and asymptotically approximates $\chi_{1}^{2}$ under the null. The second, $S_{\mathrm{Het}}$ tests for the presence of heterogeneity akin to the *Q*-test of heterogeneity and asymptotically approximates a 50:50 mixture of 0 and $\chi_{1}^{2}$ when the number of studies in a meta-analysis is large, for smaller meta-analyses, Lee *et al.* (2017) provide tabulated empirical distributions of $S_{\mathrm{Het}}$. For each $S_{\mathrm{FE}}$, the RE2C approach searches for $S_{\mathrm{Het}}$ such that $P_{\mathrm{RE}2}\;\leq \;P_{\mathrm{FE}}$ and the resulting lower boundary of $S_{\mathrm{Het}}$ is referred to as, $S_{\mathrm{Het}.\mathrm{low}}(S_{\mathrm{FE}},\;N)$ where N is the number of studies. Then for an observed RE2C statistic, $\hat{S_{\mathrm{RE}2C}}$ the range of $S_{\mathrm{FE}}$ is divided into *K* small bins $\left(x_{i}\;=\;1,\;2,\;3,\ldots ,\;K\right)$ (e.g. 1000 bins in [0, 50]) and the RE2C summary association *P*-value is approximated by:

$$P_{\mathrm{RE}2C}\;\approx \;\sum_{i=1}^{k}P\;\left(S_{\mathrm{Het}}>\max\left(\hat{S_{\mathrm{RE}2C}}-\;x_{i},\;S_{\mathrm{Het}.\mathrm{low}}\left(x_{i},\;N\right)\right)\right)\cdot \chi_{1}^{2}\left(x_{i}\right)\cdot \Delta x,$$

such that, $P_{\mathrm{RE}2C}<\;P_{\mathrm{RE}2}$ while $P_{\mathrm{RE}2}\;\leq \;P_{\mathrm{FE}}$ and where Δx denotes the width of the bins (Lee *et al.*, 2017).

### 2.3 Evaluation of heterogeneity for individual variants and *M* statistics

#### 2.3.1 Calculation of *SPRE* statistics

Standardized predicted random-effect statistics are precision-weighted residuals that capture the direction and extent with which individual genetic effects of studies in a meta-analysis deviate from the average genetic effect at a variant of interest. Consider a genetic association meta-analysis (*P*), comprising *S* GWAS (s = 1, 2, 3,…, S) and *V* independently associated lead variants (v = 1, 2, 3,…, V). At each lead variant, study effect-size estimates (and the corresponding standard errors) are analysed with a RE to estimate the average genetic effect and separate the variability observed among study effects into random sampling variation and between-study heterogeneity. A *SPRE* is then computed for each lead variant such that the *SPRE* for the *v*th lead variant in the *s*th study is:

(4) $$\mathrm{SPR}E_{\mathrm{sv}}=\frac{y_{\mathrm{sv}}-\;\theta_{v}}{\sqrt{\sigma_{\mathrm{sv}}^{2}+\;{\hat{\tau }}_{v}^{2}-\;s_{p_{\mathrm{sv}}}^{2}}}\;$$

This yields an array of *SPRE*s,

(5) $$P_{S,\;V}=\;\left[\begin{array}{cccc} \;\mathrm{SPR}E_{1,\;1} & \;\mathrm{SPR}E_{1,\;2} & \;\cdots & \;\mathrm{SPR}E_{1,\;v}\\ \;\mathrm{SPR}E_{2,\;1} & \;\mathrm{SPR}E_{2,\;2} & \;\cdots & \;\mathrm{SPR}E_{2,\;v}\\ \;\vdots \; & \;\vdots \; & \;\ddots & \;\vdots \;\\ \;\mathrm{SPR}E_{s,\;1} & \;\mathrm{SPR}E_{s,\;2} & \;\cdots & \;\mathrm{SPR}E_{s,\;v} \end{array}\right]\;$$

that can be exploited to reveal systematic genetic differences among studies in the meta-analysis. Specifically, *SPRE*s can be aggregated by study to expose outlier studies showing either consistently stronger or weaker than average genetic effects.

#### 2.3.2 Calculation of *M* statistics—aggregation of *SPRE*s

*SPRE* statistics can be aggregated in a variety of ways, a simple approach that both identifies systematic outliers and reveals their direction of effect is to calculate the ‘mean’ aggregate heterogeneity statistic, *M*. *M* statistics are computed by calculating the arithmetic mean of *SPRE*s within each study in a meta-analysis so that each study has a single *M* statistic value and the *M* statistic value for the *s*th study is represented by:

(6) $$M_{s}=\;\frac{1}{V}\sum_{v=1}^{V}{\mathrm{SPRE}}_{\mathrm{sv}}\;.$$

Assuming the *SPRE*s of lead variants in each study are mutually independent standard normal random variables, that is

$$\mathrm{SPRE}\;∼\;\Phi \left(0,\;1\right),$$

with mean: $E\left(\mathrm{SPRE}\right)=\;0$ and variance: $\mathrm{Var}\left(\mathrm{SPRE}\right)=\;1$ then *M* is normally distributed,

$$M_{s}\;∼\;\Phi \left(0,\;\frac{1}{V}\right),$$

with mean: $E\left(M_{s}\right)=\;V\times \;\left(\frac{1}{V}\;\right)\times \;\mu \;\;=\;0$ and variance: $\mathrm{Var}\left(M_{s}\right)=\;V\times \;({\frac{1}{V})}^{2}\;\times \;\sigma^{2}\;=\;\frac{1}{V}$.

#### 2.3.3 Q-statistic and heterogeneity index

Heterogeneity was also assessed using the Q-statistic (Cochran, 1954) and the heterogeneity index (*I*^2^) measure (Higgins and Thompson, 2002); *I*^2^ was further used to quantify heterogeneity in *M* statistics.

## 3 Results

### 3.1 RE2C association analysis

Of 9 455 778 variants in a RE2C meta-analysis of 48 CARDIoGRAMplusC4D studies, 4645 showed genome-wide significant associations with coronary disease (*P*_RE2C_<5×10^−8^), yielding 382 loci where lead variants were centered on a genetic distance window of ± 0.5 cM (Table 1).

**Table 1.** A summary of RE2C association results from the CARDIoGRAMplusC4D meta-analysis of coronary disease

Description	
Number of variants examined in the CARDIoGRAMplusC4D meta-analysis of coronary disease	9 455 778
Number of variants significantly associated with coronary disease under the RE2C method (*P*_RE2C_<5×10^−8^)	4645
Number of loci obtained after grouping the 4645 significantly associated variants by a genetic distance window of ±0.5 cM around each lead variant	382
Number of lead variants that replicated in the UK Biobank (UKBB) prospective study (*P*_UKBB_<5×10^−5^)	24

cM, centiMorgans.

**Fig. 1.**
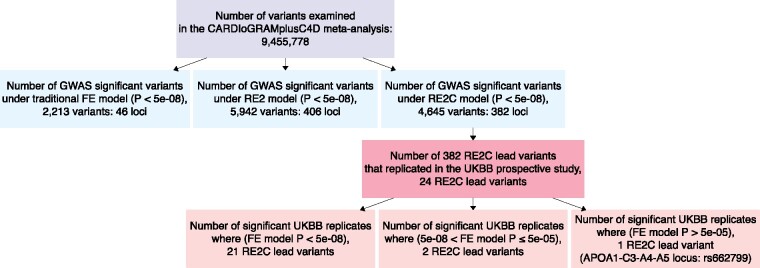
A flowchart summarizing meta-analysis genetic association results under the RE2 and RE2C random-effects models and the traditional fixed-effect (FE) method

This compares with the conventional FE meta-analysis that revealed 2213 GWAS (*P*_FE_<5×10^−8^) variants in 46 loci, and an RE2 analysis that afforded 5942 GWAS (*P*_RE2_<5×10^−8^) variants in 406 loci (Fig. 1).

### 3.2 Single-variant heterogeneity analysis of 382 novel RE2C loci

Most (85.6%) of the lead variants showed marked heterogeneity (Q-statistic *P *<* *1×10^−7^), with at least half of the lead variants showing relatively high levels of heterogeneity (*I*^2^>72.1%) (Supplementary Table S2). Next, we calculated *SPRE*s and generated forest plots to inspect heterogeneity patterns at lead variants of the 382 RE2C loci. Most (90%) of the RE2C lead variants had one or more outlier studies where genetic effect-size estimates deviated substantially $\left(\left|\mathrm{SPRE}\right|\;>\;3\sigma \right)$ from the average genetic effect. This empirical threshold to flag overly influential outliers $\left(\left|\mathrm{SPRE}\right|\;>\;3\sigma \right)$ was informed by rs2891168 (chromosome 9p21) in the primary CARDIoGRAMplusC4D meta-analysis, where this well-established locus had max $\left|\mathrm{SPRE}\right|=2.87$ (CARDIoGRAMplusC4D Consortium, 2015). An inspection of forest plots for the 382 RE2C lead variants revealed heterogeneity patterns that were grouped into three categories (Supplementary Fig. S1). Most (*n* = 323) of the lead variants fell in the first category where at least one study showed outsized effects while the majority of the studies showed minimal effects (Supplementary Fig. S2 and Table S3). Lead variants (*n* = 28) in the second category generally showed heterogeneity patterns with outlier studies showing contrasting effects, in particular the forest plots showed both positive outlier studies (SPRE > +3σ) with the potential to inflate the average genetic effect as well as negative outliers (SPRE< -3σ) that might lower or change the direction of the mean genetic effect, a scenario where dropping either type of outlier would likely induce a false positive or negative signal (Supplementary Fig. S3 and Table S3). The final category comprised 31 lead variants where there was little evidence of overly influential outlier studies consistent with heterogeneity patterns plausibly induced by biologically relevant differences (Supplementary Fig. S4 and Table S3). A general trend that emerged from inspecting heterogeneity patterns at the individual RE2C lead variants was that RE2C *P*-values became more extreme (i.e. smaller) with increasing levels of heterogeneity (Supplementary Table S2).

### 3.3 *M* Statistic, multi-variant heterogeneity analysis

A multi-variant heterogeneity analysis across the 382 RE2C lead variants revealed five significant outlier studies (14, 15, 16, 17 and 18) that systematically showed stronger than average effects (Bonferroni-corrected *M* statistic *P*-values <0.05) (Supplementary Fig. S5). A meta-regression of the *M* statistics found no evidence of systematic heterogeneity patterns due to differences in ancestry, age-of CAD onset and CAD family-history (Supplementary Table S4), design factors that were prominent in our previous analysis of the CARDIoGRAMplusC4D data (Magosi *et al.*, 2017) using lead variants for 46 published loci (CARDIoGRAMplusC4D Consortium, 2015). We note that studies 15, 16, 17 and 18 showed relatively high genomic inflation (1.08<λ < 1.38) prior to study-level genomic correction and a meta-regression of the *M* statistics confirmed varying levels of genomic inflation among contributing studies in the CARDIoGRAMplusC4D meta-analysis as a significant explanatory factor ($F_{4,\;43}=16.68,\;P\;=\;2.52\;\times \;10^{-8},\;\text{adjusted}-R^{2}=70.55\%,\;I^{2}=88.28\%$) (Supplementary Table S4).

### 3.4 Replication in the UK Biobank study

We next explored whether genetic associations between lead variants at the novel RE2C loci and CAD risk could be replicated in a large-scale prospective study based on 296 525 participants (including 34 541 cases of coronary heart disease) from England, Scotland and Wales aged 45–69 years (van der Harst and Verweij, 2018). Only 24 of the 323 RE2C lead variants available in the UK Biobank GWAS were replicated (*P*_UKBB_ < 5 × 10^−5^, Supplementary Table S5). All but 3 of the replicated genetic signals had traditional FE meta-analysis *P*-values that were significant at genome-wide levels ($P_{\mathrm{FE}}$ < 5 × 10^−8^) and just 2 of the 24 showed marked heterogeneity (*I*^2^>0.5) (Supplementary Table S5). Furthermore, 3 replicated variants included an influential outlier study in the CARDIoGRAMplusC4D meta-analysis, these 3 variants were also GWAS-significant in the FE meta-analysis (Supplementary Tables S2 and S5). These findings are consistent with Han and Eskin’s (2011) observation that the power of RE2 only exceeded FE meta-analysis for markedly heterogeneous variants.

Finally, a meta-regression model of *M* statistics for 323 RE2C lead variants in a combined CARDIoGRAMplusC4D and UK Biobank meta-analysis confirmed genomic control inflation as a potential source of systematic heterogeneity in genetic meta-analyses (Supplementary Table S6 and Fig. S6).

## 4 Discussion

Our application of the RE2C method to the CARDIoGRAMplusC4D meta-analysis dataset highlights the high sensitivity but low specificity of the method as a discovery tool for small-effect heterogeneous genetic associations. Consequently the practical advantage afforded by the improved power of the RE2C method will likely be in augmenting *P*-values for putative loci highlighted by traditional fixed and random-effects meta-analyses.

Beyond variants that would have otherwise been detected through a traditional FE meta-analysis approach, 21 lead variants that were associated with CAD under the RE2C method (*P*_RE2C_ < 5 × 10^−8^) were suggestively associated under the traditional FE method (5×10^−8^ < $P_{\mathrm{FE}}\;\leq$ 5 × 10^−5^); and 2 (rs12509595, rs62181365) of these were part of the group of RE2C lead variants that replicated in the UKBB analysis while the remaining 19 fell below the replication threshold (*P*_UKBB_<5×10^−5^) (Supplementary Tables S2 and S5). Of the list of 24 significant RE2C replicated variants in the UKBB analysis, a single lead variant (rs662799) on chromosome 11 showed neither significant nor suggestive association with CAD under the traditional FE method (Q-statistic *P *=* *2.4×10^−4^, *I*^2^ = 47%, *P*_FE_ = 1.28×10^−4^) (Supplementary Table S4 and Fig. S7). Notably, rs662799 maps to the *APOA1-C3-A4-A5* locus, immediately upstream of *APOA5*, a locus that is strongly associated with higher triglyceride levels (TG) and lower HDL cholesterol (HDL-C) in individuals of East Asian and European ancestry ($\beta_{\mathrm{TG}}=0.081,\;\mathrm{SE}(\beta_{\mathrm{TG}})=0.003,\;P\;=\;4.18\;\times \;10^{-213};\;\beta_{\mathrm{HDL}-C}=-2.516,\;{\mathrm{SE}(\beta }_{\mathrm{HDL}-C})=0.126,\;P\;=\;1.84\;\times \;10^{-85}$) (Lu *et al.*, 2016; Spracklen *et al.*, 2017). *APOA5* is a ‘well-known’ CAD-associated locus (e.g. rs964184; CARDIoGRAM Consortium *et al.*, 2011), thus the rs662799 CAD association detected in this RE2C analysis represents a confident positive assignment that can guide future functional genomic experiments to identify the underlying causal variants(s).

Altogether, the majority (*n* = 331) of lead variants discovered in the CARDIoGRAMplusC4D meta-analysis by the RE2C random-effects method fell outside the scope of tentatively associated CAD risk variants ($P_{\mathrm{FE}}$>5×10^−5^) (Supplementary Table S2). Significant *P*-values under the RE2 and RE2C models can represent a non-null average genetic effect and/or considerable heterogeneity $\left(H_{0}:\;\mu \;=\;0\;\mathrm{and}\;\tau^{2}=0\;\mathrm{versus}\;H_{1}:\;\mu \;\neq \;0\;\mathrm{or}\;\tau^{2}>0;\;\mathrm{asymptotically}\right)$ (Neupane *et al.*, 2012). Therefore, the genome-wide significant RE2C *P*-values at the 277 lead variants where genetic associations with CAD were irreproducible in the UKBB dataset (*P*_UKBB_>5×10^−5^) and where $P_{\mathrm{FE}}$>5×10^−5^, likely signify substantial heterogeneity of genetic effects at the individual variants rather than novel CAD signals.

Small-effect genetic associations at variants with relatively high heterogeneity might elicit skepticism regarding the potential reproducibility of such associations. However, there are notable exceptions within the coronary disease landscape, such as rs2891168, the lead variant for the chromosome 9p21 CAD risk locus in the CARDIoGRAMplusC4D data (2015) that shows substantial heterogeneity (Q-statistic *P *<* *4.2×10^−7^; $I^{2}=\;58\%$) but with no exceptional outlier studies (i.e. $\left|\mathrm{SPRE}\right|<\;2.6\sigma$), a heterogeneity pattern typified in Supplementary Figure S4. rs2891168 tags one of the strongest associated loci in CARDIoGRAMplusC4D (odds ratio = 1.2, *P *<* *2×10^−98^), a meta-analysis dataset heavily weighted by European (69%), South Asian (20%) and East Asian (7%) data (Supplementary Table S1). Other tagging variants for this locus in strong linkage disequilibrium have been convincingly validated to show comparable strength associations with CAD risk in some non-European populations (e.g. India and Pakistan, Coronary Artery Disease (C4D) Genetics Consortium, 2011; Han Chinese, Lu *et al.*, 2012; multi-ethnic cohorts from East Asia, Han *et al.*, 2017) but not for instance, and to our knowledge in populations of African ancestry. The latter are poorly represented in CARDIoGRAMplusC4D (African Americans form ∼1% of the total data), limiting opportunities to judge the informativity or otherwise of individual loci in this meta-analysis dataset.

Based on our experience of applying RE2C to the CARDIoGRAMplusC4D dataset, we recommend as best practice that reports of small-effect heterogeneous loci discovered with this method be accompanied by forest plots and *SPRE* statistics to explore the distribution of genetic effect estimates across participating studies. This can highlight overly influential outlier studies with the potential to inflate genetic signals prompting researchers to reflect upon the underlying data that gave rise to novel heterogeneous associations.

## Supplementary Material

btz590_Supplementary_Data
