# Supplementary material for: Identifying small-effect genetic associations overlooked by the conventional fixed-effect model in a large-scale meta-analysis of coronary artery disease
Source: Bioinformatics. 2019 Jul 27;36(2):552–7. doi: 10.1093/bioinformatics/btz590 (PMC7223261; doi:10.1093/bioinformatics/btz590)
Supplement: btz590_Supplementary_Data [file btz590_supplementary_data.zip › btz590-Suppl_data/Supplementary_Data.pdf]

Supplemental Figures

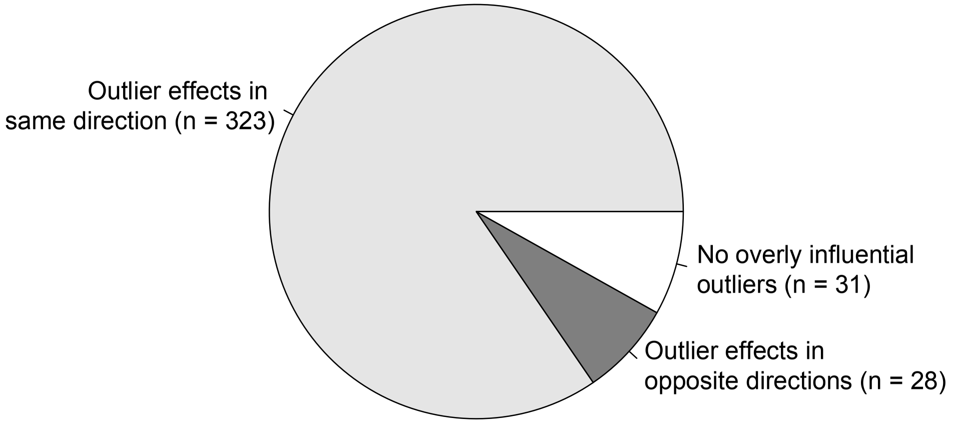

Figure S1. A pie chart classifying the 382 RE2C Han-Eskin lead variants by heterogeneity pattern. The RE2C lead variants are grouped into three categories namely: 1) lead variants where genetic effects of outlier studies were in the same direction (n = 323), 2) lead variants where outlier study-effects were in opposite directions (n = 28) and 3) lead variants where there was little evidence of overly influential outlier studies (n = 31).

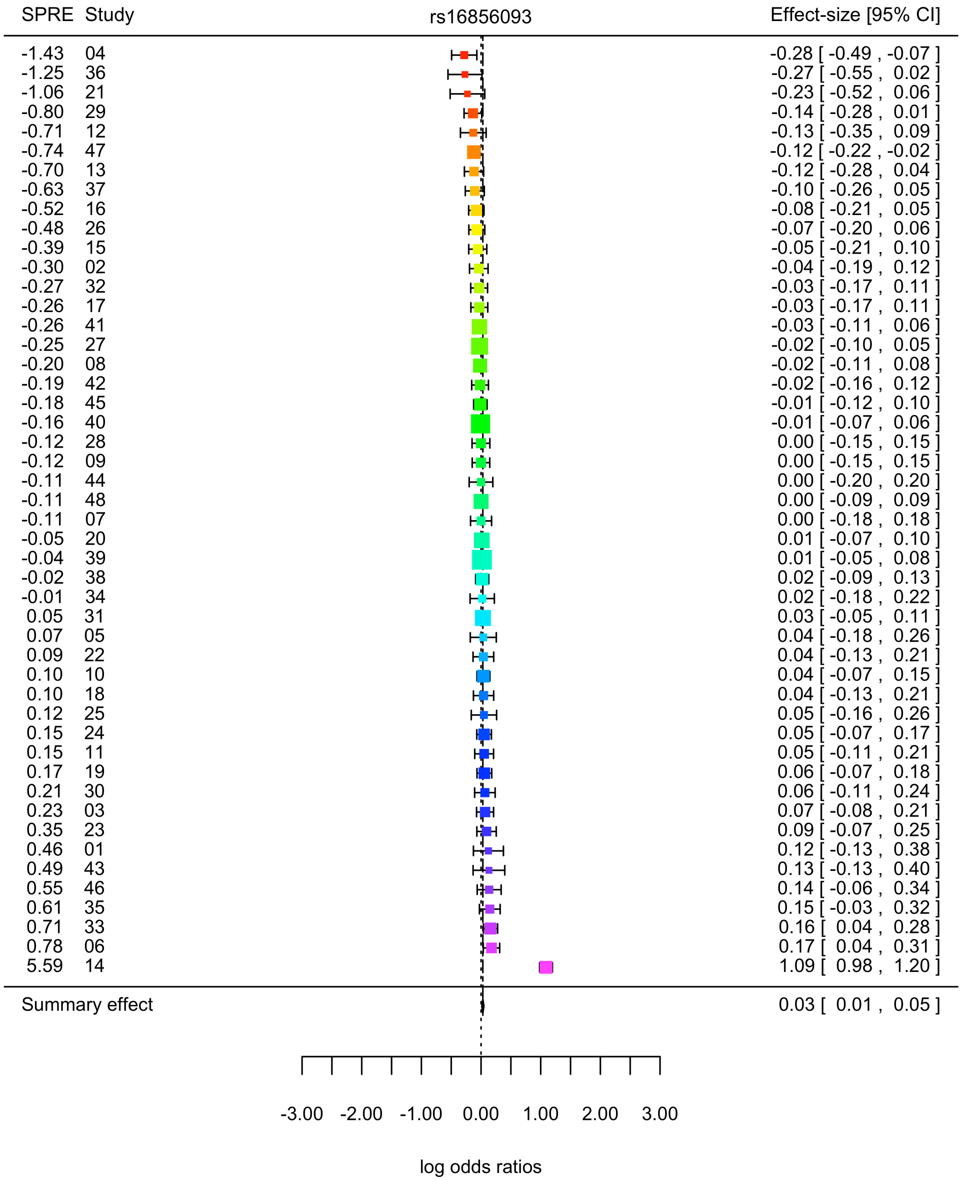

Figure S2. Forest plot of effect-size estimates (log odds ratios) at rs16856093 on chromosome 3 highlighting locus specific heterogeneity induced by a single outlier study (14) showing outsized effects in the CARDIoGRAMplusC4D meta-analysis of coronary disease (I^2 = 89.35%). Sorted log odds ratios are presented for individual studies represented by filled squares with their 95% confidence intervals shown by horizontal lines; the sizes of the squares are proportional to each studies' inverse-variance weighting. Furthermore, a colour gradient corresponding to individual studies' *SPRE* (standardized predicted random-effects) statistics is shown to highlight the deviation of each studies' effect-size estimate from the average genetic effect. A filled diamond denotes the average effect-size.

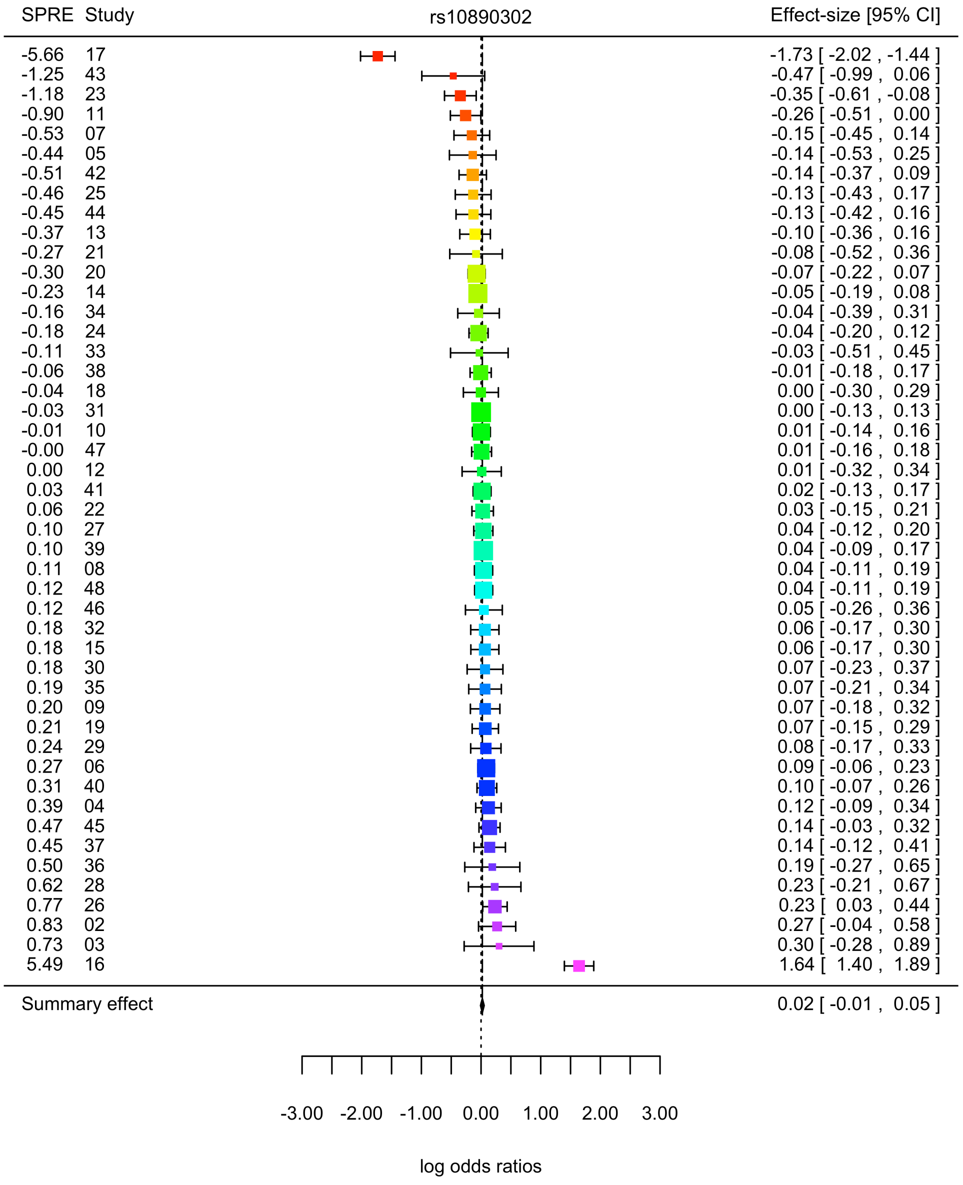

Figure S3. Forest plot of effect-size estimates (log odds ratios) at rs10890302 on chromosome 1 highlighting locus specific heterogeneity induced by outlier studies (16 and 17) showing outsized effects in opposite directions (I^2 = 86.84%) in the CARDIoGRAMplusC4D meta-analysis of coronary disease. Sorted log odds ratios are presented for individual studies represented by filled squares with their 95% confidence intervals shown by horizontal lines; the sizes of the squares are proportional to each studies' inverse-variance weighting. Moreover, a colour gradient corresponding to individual studies' *SPRE* (standardized predicted random-effects) statistics is shown to highlight the deviation of each studies' effect-size estimate from the average genetic effect. A filled diamond denotes the average effect-size.

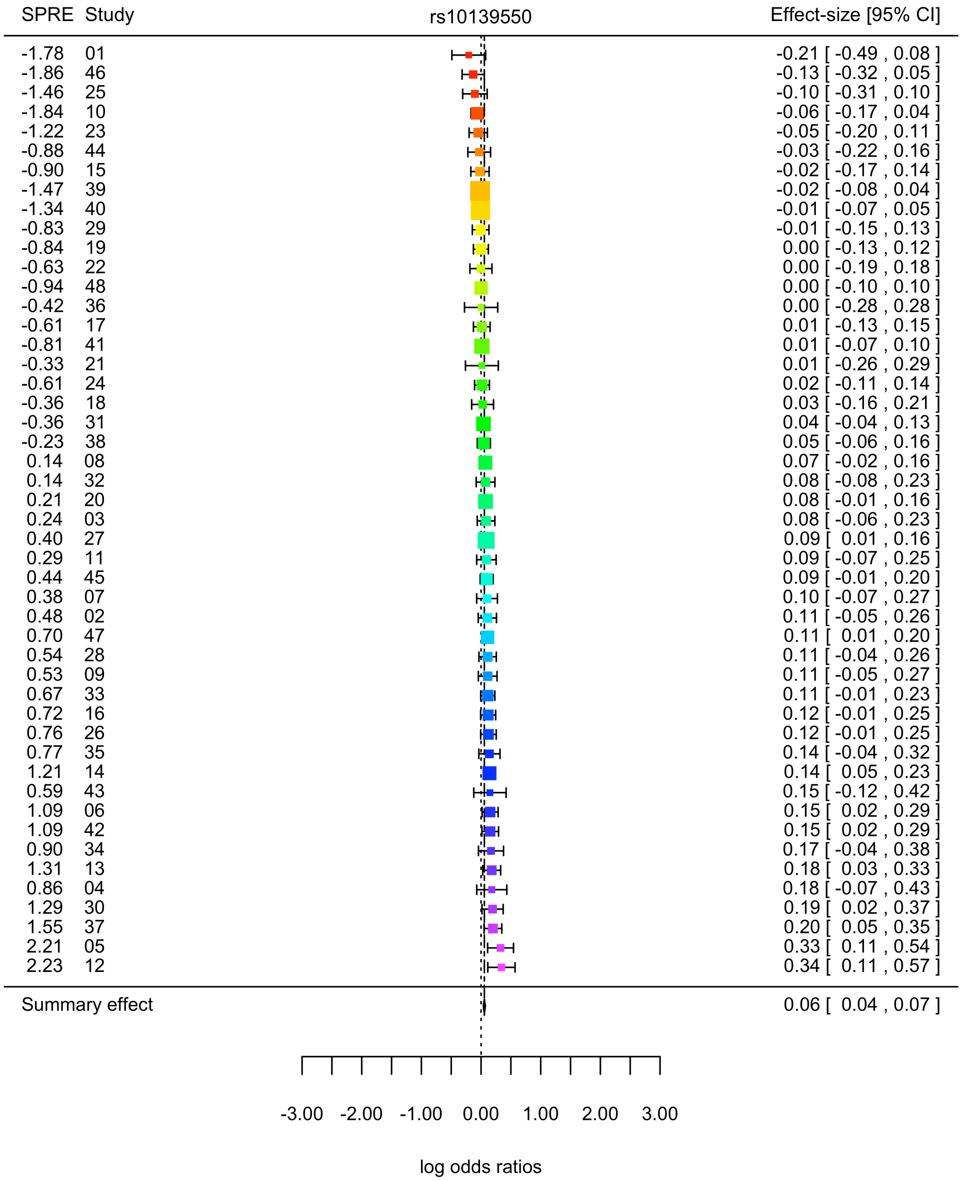

Figure S4. Forest plot of effect-size estimates (log odds ratios) at rs10139550 on chromosome 14 highlighting locus specific heterogeneity among studies in the CARDIoGRAMplusC4D meta-analysis of coronary disease in the absence of overly influential outlier studies (I^2 = 34.83%). Sorted log odds ratios are presented for individual studies represented by filled squares with their 95% confidence intervals shown by horizontal lines; the sizes of the squares are proportional to each studies' inverse-variance weighting. Furthermore, a colour gradient corresponding to individual studies' *SPRE* (standardized predicted random-effects) statistics is shown to highlight the deviation of each studies' effect-size estimate from the average genetic effect. A filled diamond denotes the average effect-size.

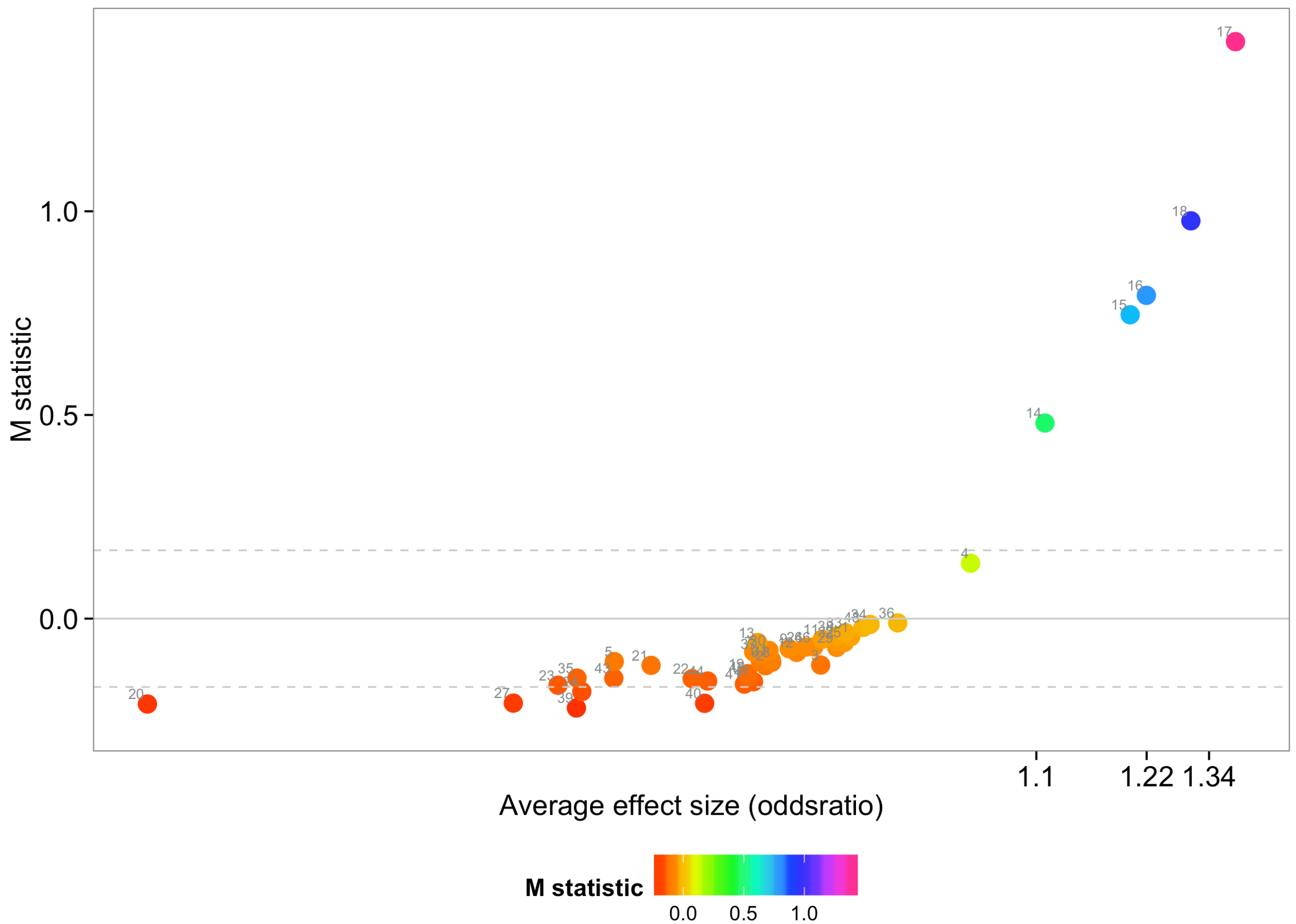

Figure S5. A scatterplot of *M* statistics computed across 382 RE2C loci. *M* statistics for each study in the CARDIoGRAMplusC4D meta-analysis (Y- axis) are plotted against the average variant effect size (expressed as odds ratios) (X-axis) in each study. A colour gradient was employed to highlight the distribution of *M* statistics. The dashed lines indicate the Bonferroni corrected 5% significance threshold ($M = \pm 0.1678$) to allow for multiple testing of 48 studies.

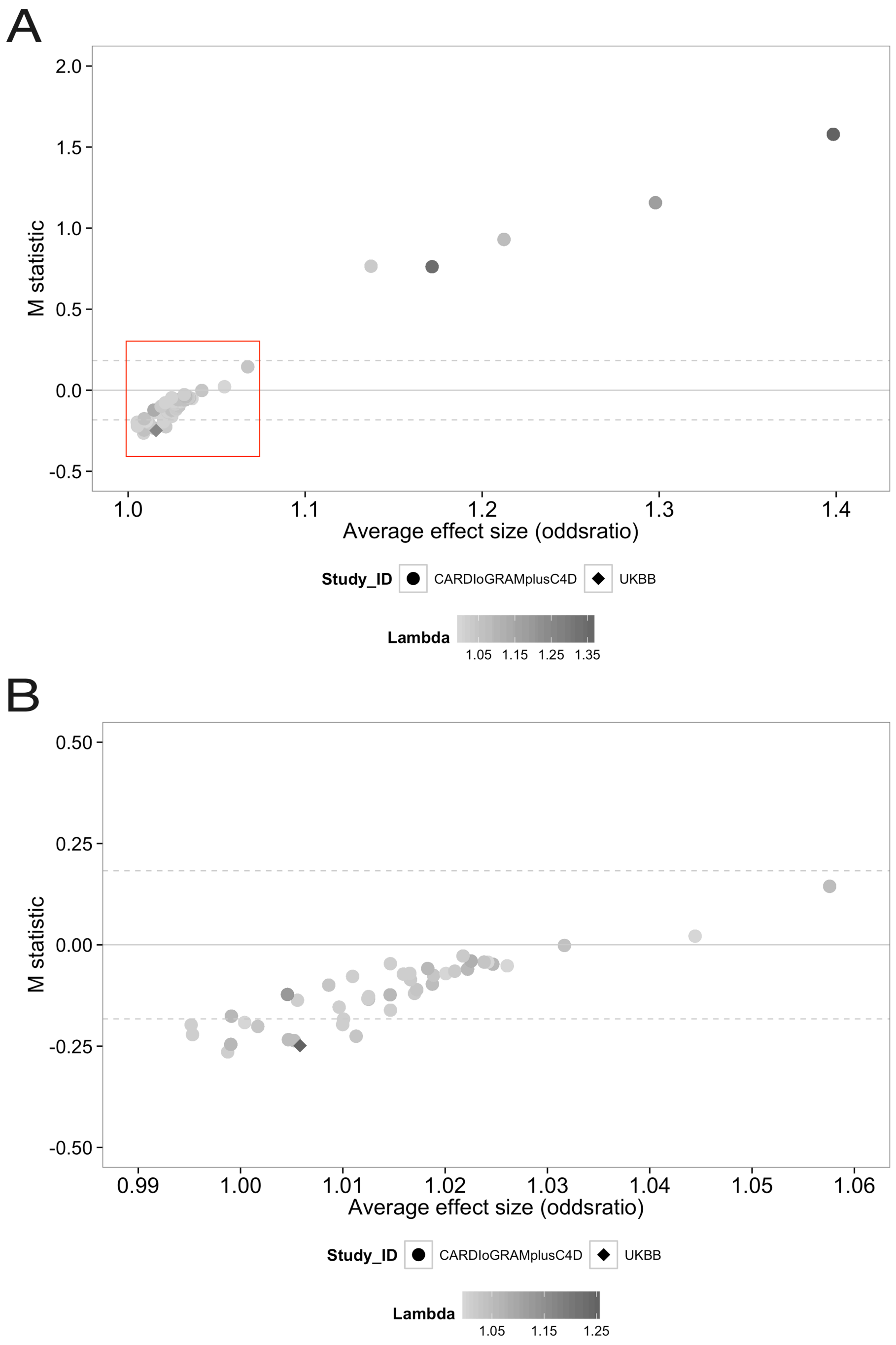

Figure S6. Scatterplots of *M* statistics computed across 323 RE2C lead variants highlighting systematic heterogeneity among the 48 studies in the CARDIoGRAMplusC4D meta-analysis and the UKBB study. *M* statistics of each of the 49 studies (Y- axis) are plotted against the average variant effect size (expressed as odds ratios) (X-axis) in each study. The distribution of genomic inflation values (lambda) among the studies is highlighted by a grey colour gradient. The dashed lines indicate the Bonferroni corrected 5% significance threshold ($M = \pm 0.1828$) to allow for multiple testing of 49 studies. Studies showing systematically stronger than average effects were mostly those with larger genomic inflation values as shown in panel A. Panel B is a zoom-in of the area highlighted by the solid red rectangle in Panel A, showing the distribution of *M* statistics in studies largely showing average genetic effects.

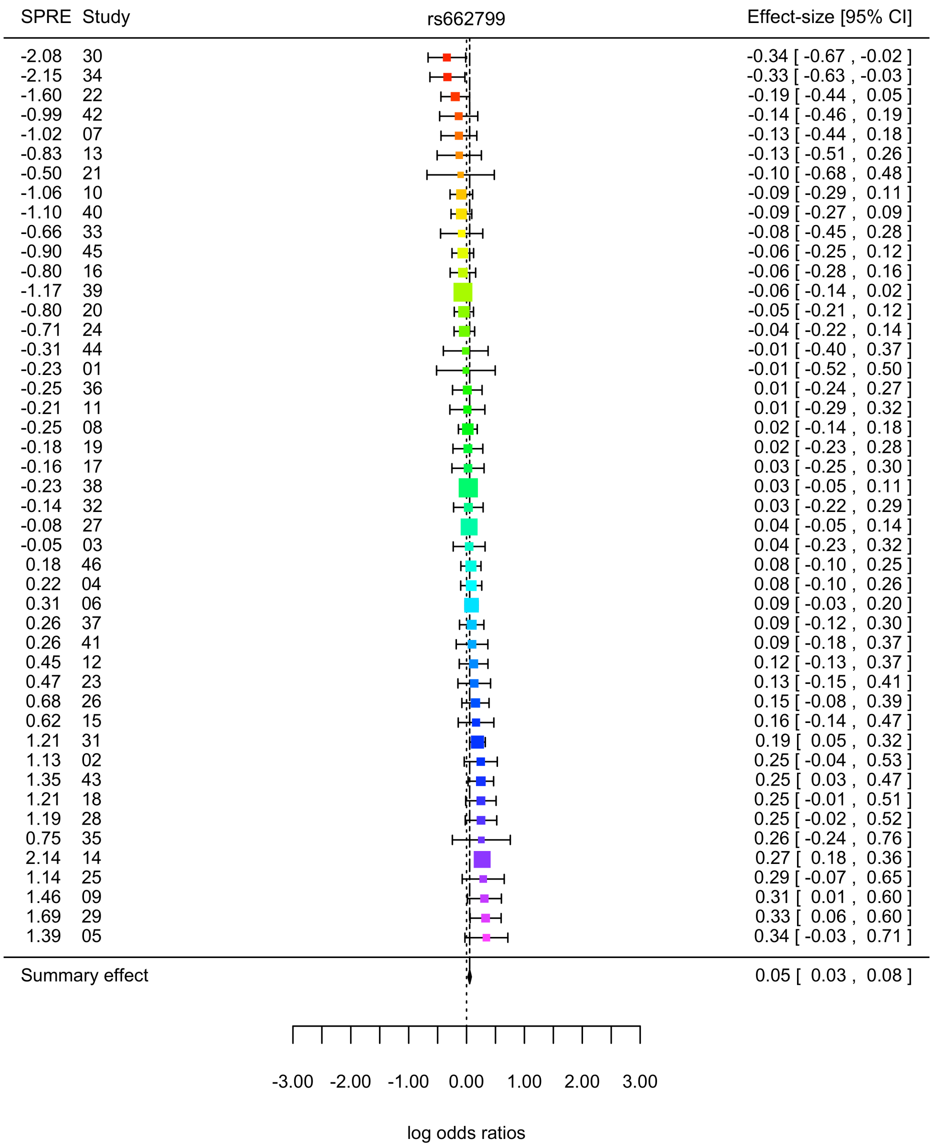

Figure S7. Forest plot highlighting the distribution of genetic effects (log odds ratios) among studies in the CARDIoGRAMplusC4D meta-analysis at rs662799 on chromosome 11. Sorted log odds ratios are presented for individual studies represented by filled squares with their 95% confidence intervals shown by horizontal lines; the sizes of the squares are proportional to each studies' inverse-variance weighting. Furthermore, a colour gradient corresponding to individual studies' *SPRE* (standardized predicted random-effects) statistics is shown to highlight the deviation of each studies' effect-size estimate from the average genetic effect. A filled diamond denotes the average effect-size.
